# ChatGPT’s Limitations in Athlete ECG Interpretation: Evidence from a Multicenter Diagnostic Study

**DOI:** 10.3390/jcdd13050191

**Published:** 2026-04-29

**Authors:** Stefano Palermi, Marco Vecchiato, Tommaso Remo Iacovone, Matteo Anselmino, Rachele Adorisio, Alessandro Biffi, Francesco Borrelli, Erica Brugin, Nicoletta Cantarutti, Elena Cavarretta, Mattia Cominacini, Marco Corsi, Flavio D’Ascenzi, Vittorio De Feo, Giuseppe Di Gioia, Gianluigi Dorelli, Giulia Foccardi, Sabina Gallina, Silvia Giangrandi, Francesca Graziano, Elisa Lodi, Alberto Livio, Viviana Maestrini, Guglielmo Leonardo Manfredi, Davide Mansour, Maria Grazia Modena, Daniel Neunhaeuserer, Antonia Nigro, Andrea Palermi, Alessio Pellegrino, Antonio Pelliccia, Filippo Maria Quattrini, Fabrizio Ricci, Fiammetta Scarzella, Maria Rosaria Squeo, Riccardo Tonelli, Emanuele Zanardo, Alessandro Zorzi, Fabrizio D’Ascenzo, Gaetano Maria De Ferrari, Andrea Saglietto

**Affiliations:** 1 Department of Medicine and Surgery, UniCamillus Saint Camillus International University of Health Sciences, 00131 Rome, Italy; tommasoriacovone01@outlook.it; 2 Sports and Exercise Medicine Division, Department of Medicine, University of Padova, 35122 Padova, Italy; marcovecchiato.md@gmail.com (M.V.); alberto.livio@studenti.unipd.it (A.L.); daniel.neunhaeuserer@unipd.it (D.N.); emanuele.zanardo@studenti.unipd.it (E.Z.); 3 Division of Cardiology, Cardiovascular and Thoracic Department, Città della Salute e della Scienza di Torino Hospital, 10126 Turin, Italy; matteo.anselmino@unito.it (M.A.); fabrizio.dascenzo@gmail.com (F.D.); gaetano.deferrari@unito.it (G.M.D.F.); andrea.saglietto@live.com (A.S.); 4 Department of Medical Sciences, University of Turin, 10124 Turin, Italy; 5 Bambino Gesù Children’s Hospital, IRCCS, 00165 Rome, Italy; rachele.adorisio@opbg.net (R.A.); nicoletta.cantarutti@opbg.net (N.C.); elena.cavarretta@uniroma1.it (E.C.); 6 Med-Ex, Medicine & Exercise, Medical Partner Scuderia Ferrari, 00187 Rome, Italy; a.biffi@libero.it; 7 Sports Medicine Institute, 10143 Turin, Italy; francesco.borrelli.1991@gmail.com (F.B.); fiammetta.scarzella@imsto.it (F.S.); 8 Sports and Exercise Medicine Division, Department of Medical Specialties, ULSS 3 Serenissima, 30174 Venice, Italy; erica.brugin@aulss3.veneto.it (E.B.); giulia.foccardi@gmail.com (G.F.); 9 Department of Medical-Surgical Sciences and Biotechnologies, Sapienza University of Rome, 04100 Latina, Italy; 10 Department of Engineering for Innovation Medicine, University of Verona, 37100 Verona, Italy; mattia.cominacini@univr.it; 11 Sports Medicine Center, University of Florence, 50100 Florence, Italy; marco.corsi@unifi.it (M.C.); alessio.pellegrino@unifi.it (A.P.); 12 Sports Cardiology and Rehab Unit, Department of Medical Biotechnologies, University of Siena, 53100 Siena, Italy; dascenzi2@unisi.it (F.D.); silvia.giangrandi@gmail.com (S.G.); guglielmo.manfredi.4@gmail.com (G.L.M.); 13 M.d.S. srl, 65100 Pescara, Italy; info@mdspescara.it; 14 University Cardiology Division, SS Annunziata Polyclinic University Hospital, 66100 Chieti, Italy; sabina.gallina@unich.it (S.G.); davidemansour@virgilio.it (D.M.); andrea.palermi@outlook.com (A.P.); fabrizio.ricci@unich.it (F.R.); 15 Institute of Sport Medicine and Science, Italian National Olympic Committee, Largo Piero Gabrielli, 00197 Rome, Italy; dottgiuseppedigioia@gmail.com (G.D.G.); viviana.maestrini@uniroma1.it (V.M.); ant.pelliccia@gmail.com (A.P.); mariarosaria.squeo@coni.it (M.R.S.); 16 Department of Neurosciences, Biomedicine and Movement Sciences, University of Verona, 37100 Verona, Italy; gianluigi.dorelli@univr.it; 17 Department of Cardiac, Thoracic and Vascular Sciences and Public Health, University of Padua, 35128 Padua, Italy; francesca.graziano@unipd.it (F.G.); riccardotonelli97@gmail.com (R.T.); alessandrozorzi@gmail.com (A.Z.); 18 Centro PASCIA (Programma Assistenziale Scompenso Cardiaco, Cardiopatie dell’Infanzia e A rischio), AOU Policlinico di Modena, 41121 Modena, Italy; elisalodi@unimore.it (E.L.); mariagrazia.modena@unimore.it (M.G.M.); 19 Villa Stuart Sport Clinic, FIFA Medical Center of Excellence, 00135 Rome, Italy; antonianigro@tiscali.it; 20 Health Promotion, Prevention Plans and Sports Medicine Unit, Department of Prevention, ASL Roma 2, 00159 Rome, Italy; filippomaria.quattrini@aslroma2.it

**Keywords:** athletes, ECG, artificial intelligence, ChatGPT, sports cardiology

## Abstract

Background: Artificial intelligence (AI) has shown promise in the interpretation of electrocardiograms (ECGs) using signal-based deep learning models. In parallel, large language models (LLMs) have gained increasing visibility in clinical practice, including exploratory applications in ECG analysis. Whether a general-purpose LLM can meaningfully discriminate cardiovascular disease from athlete ECGs during PPS remains unknown. We aimed to evaluate the diagnostic performance of a general-purpose LLM for this task. Methods: In this multicentre diagnostic accuracy study, we evaluated a commercially available LLM (ChatGPT, version 5) in 2950 competitive athletes undergoing PPS. All athletes underwent resting 12-lead ECG, with second- and third-line investigations performed when clinically indicated. The reference outcome was confirmed cardiovascular disease after full diagnostic work-up (n = 450, 15.3%). For each ECG, the LLM generated a numeric score (0–100) representing the inferred likelihood of underlying disease using a standardized prompt and without task-specific fine-tuning. Discriminative performance was assessed using receiver operating characteristic (ROC) analysis. Misclassification patterns were analysed according to International ECG Criteria. Results: GPT-derived scores demonstrated a marked floor effect, with a median value of 0 (IQR 0–2) in both diseased and non-diseased athletes and substantial overlap between groups. The area under the ROC curve was 0.52 (95% CI 0.49–0.55), indicating performance close to random classification. At the Youden-derived threshold, 79% of athletes with confirmed disease were incorrectly classified as negative. False-negative cases were predominantly characterized by borderline ECG patterns (82%), and a substantial number of red-flag ECG abnormalities were also missed. Conclusions: In this PPS cohort, a general-purpose LLM used in a naïve configuration showed no clinically meaningful ability to discriminate between cardiovascular disease and athlete ECGs. Without task-specific training or domain adaptation, such models should not be used for diagnostic triage in athlete screening.

## 1. Introduction

Cardiovascular pre-participation screening (PPS) in competitive athletes aims to identify individuals at risk of adverse cardiovascular events, including sudden cardiac death, while minimizing unnecessary downstream investigations and inappropriate sport disqualification [1]. Resting 12-lead electrocardiography (ECG) is a cornerstone of PPS in several countries, including Italy [1,2].

Despite standardized athlete-specific interpretation criteria [3], ECG evaluation in athletes remains challenging. Physiological exercise-induced cardiac remodeling may overlap with early manifestations of structural or electrical disease, particularly in borderline patterns that often require further evaluation [2,4,5]. As a result, diagnostic uncertainty remains a central issue in PPS [6].

Artificial intelligence (AI) has been increasingly proposed to improve ECG interpretation. Signal-based deep learning models trained on raw ECG waveforms have demonstrated promising performance in detecting specific cardiovascular conditions [7]. However, these models are typically task-specific and developed on controlled datasets, with limited validation in athlete populations characterized by low disease prevalence and high physiological variability [8].

In parallel, large language models (LLMs) have rapidly gained visibility in clinical practice. LLMs are probabilistic models trained on large multimodal datasets to generate and interpret human-like outputs. Recent multimodal versions can process medical images, including ECG tracings, and are increasingly used as general-purpose diagnostic assistants [9,10]. However, unlike signal-based AI models, LLMs are not specifically trained to extract structured physiological features from ECG waveforms.

Whether such general-purpose models can reliably distinguish physiological from pathological ECG patterns in athletes remains unknown. This question is clinically relevant, as these tools are widely accessible and may be used informally in real-world settings. The aim of this study was to evaluate the diagnostic performance of a general-purpose LLM in discriminating cardiovascular disease from athlete ECGs during PPS.

## 2. Materials and Methods

### 2.1. Study Design and Setting

This was a retrospective, multicentre diagnostic accuracy study evaluating the performance of a general-purpose commercially available LLM used in a naïve configuration, in discriminating cardiovascular disease from resting 12-lead ECGs obtained during PPS in competitive athletes, reflecting real-world accessibility rather than optimized or fine-tuned performance.

The study was conducted across multiple Italian sports cardiology centers participating in standardized PPS programs consistent with national recommendations [1]. The study was designed and reported in accordance with key STARD principles for diagnostic accuracy studies. This study was conducted in accordance with the principles of the Declaration of Helsinki, and was approved by the local ethical committee. The analysis was performed on fully anonymized clinical data collected during routine pre-participation screening, with no intervention or modification of clinical management.

### 2.2. Study Population and Screening Protocol

We analyzed data from 2950 competitive athletes undergoing routine PPS across participating centers. Screening included medical history, physical examination, resting 12-lead electrocardiogram (ECG), and exercise stress testing as first-line evaluation, in accordance with Italian national recommendations [1]. Athletes were competitive but not necessarily professional. All ECGs were acquired in standard 12-lead format during routine PPS.

When clinically indicated, second- and third-line investigations were performed, including transthoracic echocardiography, ambulatory ECG monitoring, cardiac magnetic resonance (CMR), computed tomography (CT), or additional diagnostic procedures according to established eligibility criteria [11,12].

The cohort reflects real-world PPS practice and was not fully consecutive with universal imaging. The reference outcome was the presence or absence of confirmed cardiovascular disease following completion of the full diagnostic work-up [1].

All cardiovascular diagnoses were established at each participating center according to current international and national guidelines, using a multimodality approach [1,2,5]. Diagnostic pathways varied according to the suspected condition but generally included integration of clinical evaluation, ECG findings, and advanced imaging techniques. For example, cardiomyopathies were defined according to contemporary consensus criteria using echocardiography and CMR [2,5]; coronary artery anomalies were identified using CT or CMR [5]; and channelopathies such as Wolff–Parkinson–White syndrome or long QT syndrome were diagnosed based on ECG criteria, with additional testing when required [3]. Inflammatory and ischemic conditions were defined using standard clinical, imaging, and laboratory criteria [5].

Importantly, outcome classification was based on the final clinical diagnosis integrating all available investigations, rather than ECG findings alone [1]. Thus, the LLM was evaluated against imaging and clinically confirmed disease rather than athlete-specific ECG interpretation.

For sensitivity analysis, cardiovascular conditions were categorized a priori according to their expected detectability from ECG. ECG-detectable conditions included pre-excitation syndromes, channelopathies, coronary artery anomalies, cardiomyopathies, myocarditis/pericarditis, non-ischemic left ventricular scar, and ischemic heart disease [2,5]. In this analysis, athletes with non-ECG-detectable conditions were excluded, and model performance was evaluated by comparing athletes with ECG-detectable disease against those without cardiovascular disease.

### 2.3. LLM Configuration and Prompt Specification

A GPT-based large language model (ChatGPT, version 5; OpenAI) was used to generate a numeric probability estimate for each ECG.

Each ECG was provided to the model in image format, in standard 12-lead layout as obtained during PPS, without accompanying clinical information, demographic data, diagnostic labels, or prior interpretation.

The following standardized prompt was used for all ECGs: “Here is an athlete’s ECG. Based on your interpretation, provide a single number between 0 and 100 indicating the likelihood that this ECG suggests underlying cardiovascular disease. Use 0 for an ECG that is certainly normal and 100 for an ECG that is certainly abnormal. Do not include text, interpretation, or narrative. Output only the number.”

No additional instructions, system prompts, temperature adjustments, calibration procedures, or task-specific fine-tuning were applied. The model was used in its commercially available configuration at the time of analysis. Each ECG was processed independently in separate sessions to avoid contextual carryover effects. If the output contained non-numeric characters, the same standardized prompt was reissued until a single numeric value was obtained.

The resulting numeric value (0–100) was recorded as the GPT-derived score and treated as a continuous predictor. This design was intentionally chosen to replicate naïve real-world use of a general-purpose LLM by clinicians rather than to optimize diagnostic performance.

### 2.4. ECG Classification According to International Criteria

According to the 2017 International Criteria, ECG findings were categorized as green, yellow, or red. Green findings were defined as physiological ECG patterns related to athletic adaptation. Yellow findings were defined as borderline patterns that may reflect either physiological adaptation or early disease and generally require further evaluation when present in combination. Red findings were defined as pathological ECG abnormalities suggestive of underlying cardiovascular disease and requiring further diagnostic work-up. This categorization was performed independently of the LLM output and was used solely for subgroup analysis to explore patterns of misclassification.

### 2.5. Statistical Analysis

Continuous variables are reported as mean ± standard deviation (SD) or median with interquartile range (IQR), as appropriate. Categorical variables are expressed as counts and percentages.

**Discrimination analysis.** The discriminative performance of the GPT-derived score was assessed using receiver operating characteristic (ROC) curve analysis. The area under the ROC curve (AUC) was calculated with 95% confidence intervals (CI) using DeLong’s method.

**Threshold analysis.** For descriptive purposes, an optimal operating threshold was identified using Youden’s J statistic (sensitivity + specificity − 1). Diagnostic performance metrics at this threshold included:

- Sensitivity;
- Specificity;
- Positive predictive value (PPV);
- Negative predictive value (NPV).

Given the screening context and relatively low disease prevalence, predictive values were interpreted in light of pre-test probability.

**Score distribution analysis.** To assess separation between diseased and non-diseased athletes, score distributions were analysed using summary statistics and graphical inspection. Particular attention was given to potential floor or ceiling effects.

**Misclassification analysis.** Among athletes with confirmed cardiovascular disease, cases were classified as:

- True positives (GPT score ≥ threshold);
- False negatives (GPT score < threshold).

The distribution of International ECG Criteria categories (Green, Yellow, Red) was analysed within these subgroups to explore whether the model preferentially identified overt ECG abnormalities while failing in borderline patterns.

**Sensitivity analysis.** For the sensitivity analysis, athletes with non-ECG-detectable cardiovascular diseases were excluded. The analysis compared athletes with ECG-detectable diseases against athletes without cardiovascular disease.

All statistical analyses were performed using R (version 4.x) with the pROC package for ROC analysis. A two-sided *p*-value < 0.05 was considered statistically significant.

## 3. Results

### 3.1. Cohort Characteristics

The study included 2950 competitive athletes undergoing PPS across participating centers. The mean age was 23 ± 9 years, and 72% were male. After completion of second- or third-line investigations, 450 athletes (15.3%) were diagnosed with a confirmed cardiovascular disease, while 2500 (84.7%) had no evidence of disease (Table 1).

The spectrum of confirmed diagnoses was heterogeneous and reflected real-world PPS practice, including electrical disorders, congenital abnormalities, structural heart disease, inflammatory conditions, ischemic heart disease, and hypertension (Table 2). Importantly, not all confirmed diseases were expected to manifest with specific ECG abnormalities.

### 3.2. Distribution of GPT-Derived Scores

**Figure 1:**
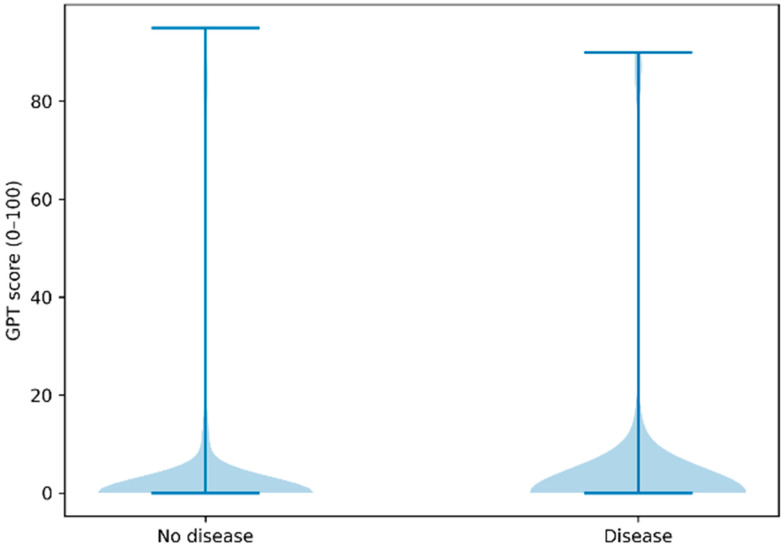
Distribution of GPT-derived scores in athletes with and without confirmed cardiovascular disease. Distribution of GPT-derived scores (0–100) among athletes with confirmed cardiovascular disease (n = 450) (on the right) and those without disease (n = 2500) (on the left). Scores ranged from 0 to 95 but demonstrated a pronounced floor effect, with a median value of 0 (interquartile range 0–2) in both groups. Substantial overlap between diseased and non-diseased athletes is observed across the entire score range, indicating no meaningful separation or probabilistic discrimination.

GPT-derived scores ranged from 0 to 95. However, a pronounced floor effect was observed. The median score was 0 (interquartile range 0–2) in both diseased and non-diseased athletes. Score distributions demonstrated substantial overlap between groups, with the majority of ECGs assigned a score of 0 or near-zero values irrespective of disease status (Figure 1).

### 3.3. Discriminative Performance

**Figure 2:**
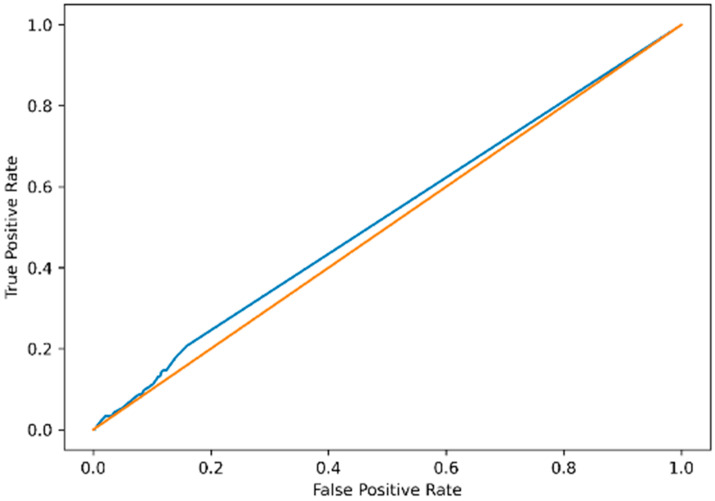
Receiver operating characteristic (ROC) curve of the GPT-derived score for the detection of cardiovascular disease. Receiver operating characteristic (ROC) curve evaluating the discriminative performance of the GPT-derived score for identifying confirmed cardiovascular disease in competitive athletes. The area under the curve (AUC) was 0.52 (95% CI 0.49–0.55) (blue), indicating performance close to random classification (orange). The ROC curve demonstrates minimal deviation from the diagonal reference line across the entire threshold spectrum.

ROC analysis demonstrated poor overall discrimination. AUC was 0.52 (95% CI: 0.49–0.55), indicating performance close to random classification (Figure 2). The ROC curve showed minimal deviation from the diagonal reference line, confirming the absence of meaningful discriminatory capacity across the entire score range.

### 3.4. Threshold-Based Classification Performance

The optimal threshold identified using Youden’s J statistic corresponded to a near-zero cut-off (GPT score ≥ 0.05). Given that model outputs were integer values between 0 and 100, this threshold effectively classified any score ≥ 1 as positive.

At this threshold:

True positives: 93/450 (20.7%)
False negatives: 357/450 (79.3%)
True negatives: 1675/2500 (67.0%)
False positives: 825/2500 (33.0%)

Diagnostic metrics at this threshold are reported in Table 3.

Sensitivity remained low across higher thresholds, while specificity increased at the cost of further reduction in sensitivity. Positive predictive values were modest across all cut-offs and largely reflected disease prevalence rather than meaningful discriminatory capacity.

### 3.5. Misclassification Analysis According to International ECG Criteria

Among the 450 athletes with confirmed cardiovascular disease:

93 were classified as true positives.

357 were classified as false negatives.

True positives were more frequently associated with pathological (red) ECG patterns (45.2%), whereas false-negative cases were predominantly characterized by borderline (yellow) ECG findings (82.4%) (Table 4).

**Figure 3:**
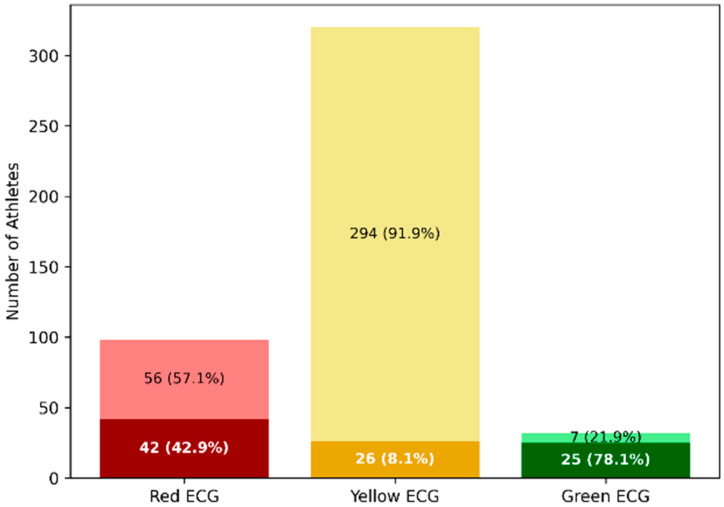
Misclassification patterns according to the 2017 International ECG Criteria in athletes with confirmed cardiovascular disease. Stacked bar chart illustrating the distribution of International ECG Criteria categories among athletes with confirmed cardiovascular disease (n = 450), stratified by GPT-based classification at the Youden-derived threshold (GPT score ≥ 0.05). Within each ECG category: Dark shades represent true-positive classifications (correctly identified disease). Light shades represent false-negative classifications (missed disease). In athletes with pathological (red) ECG patterns, 42 cases were correctly identified (dark red), whereas 56 were incorrectly classified as negative (light red). Among borderline (yellow) ECG patterns, false negatives (light yellow) predominated (294 cases), highlighting the model’s inability to discriminate subtle abnormalities. Green ECGs were infrequent among diseased athletes, yet misclassification persisted. This visualization underscores that misclassification was not limited to subtle patterns but also affected overt pathological ECG abnormalities.

Notably, 56 athletes with red-flag ECG abnormalities were incorrectly classified as negative, indicating failure to detect overt pathological patterns (Figure 3).

In a sensitivity analysis restricted to ECG-detectable cardiovascular conditions, including pre-excitation, channelopathies, coronary artery anomalies, cardiomyopathies, myocarditis/pericarditis, non-ischemic left ventricular scar, and ischemic heart disease, the GPT-derived score showed no meaningful improvement in discrimination. Among 2677 athletes included in this analysis, 177 had ECG-detectable disease and 2500 had no disease. The AUC was 0.52 (95% CI 0.49–0.55), confirming performance close to random classification.

## 4. Discussion

### 4.1. Principal Findings

In this large multicenter cohort of competitive athletes undergoing PPS, a commercially available general-purpose LLM, used in a naïve real-world configuration, demonstrated no clinically meaningful ability to discriminate athletes with confirmed cardiovascular disease from those without. The GPT-derived score exhibited a marked floor effect, substantial overlap between diseased and non-diseased athletes, and an AUC of 0.52, indicating performance close to random classification. The near-zero optimal threshold suggests that the model did not generate a meaningful probability continuum but rather a quasi-binary distribution concentrated at zero. Given that model outputs were integer values between 0 and 100, this threshold effectively classified any score ≥ 1 as positive. At the Youden-derived threshold, nearly four out of five athletes with confirmed cardiovascular disease were incorrectly classified as negative. Importantly, false-negative cases were predominantly characterized by borderline ECG patterns according to International Criteria, the very subgroup that most frequently requires expert evaluation in PPS.

### 4.2. Structural Mismatch Between LLM Architecture and Signal-Dependent Tasks

These findings likely reflect a fundamental architectural mismatch between language-based probabilistic models and signal-dependent diagnostic tasks [13,14,15].

Dedicated AI ECG systems are typically trained directly on raw waveform data using convolutional neural networks (CNNs) or related architectures optimized for temporal feature extraction [16]. Such models have demonstrated the ability to detect specific structural and functional abnormalities, including hypertrophic cardiomyopathy and left ventricular dysfunction, from ECG signals [7]. More recently, ensemble deep learning models based on ECG images have also shown promising performance for structural heart disease detection [17]. Importantly, these systems are trained on labeled cardiovascular outcomes and explicitly optimized for predefined diagnostic targets.

In contrast, general-purpose LLMs are trained to model token-level probability distributions across large multimodal corpora and are not inherently optimized for high-resolution physiological signal analysis [13]. Although multimodal variants can process ECG images, they do not perform structured extraction of time-series electrical features such as QRS duration, voltage amplitudes, repolarization morphology, or lead-specific temporal relationships in the manner of signal-trained CNNs. Their reasoning is probabilistic and context-driven rather than waveform-native.

Exploratory studies evaluating LLMs for ECG interpretation have reported variable and inconsistent performance, particularly in complex or ambiguous cases [18,19]. In a recent image-based myocardial infarction classification study, ChatGPT achieved an AUC of 0.57, with low sensitivity despite moderate specificity, indicating limited discriminative capacity even in a binary acute-care task with overt ECG abnormalities [20]. Similarly, comparative analyses between GPT-based models and cardiologists in emergency department settings have shown variable accuracy and reduced reliability in identifying repolarization abnormalities and subtle ST–T changes [21]. Multimodal evaluations of ChatGPT-4V on ECG image interpretation tasks have demonstrated reasonable performance in structured, multiple-choice formats but lower accuracy in open diagnostic inference and waveform-dependent tasks, particularly when precise morphological assessment is required [22]. Furthermore, in complex electrophysiological prediction tasks—such as the localization of ventricular ectopic foci prior to ablation—ChatGPT-4o performed no better than chance (kappa approximately 0), underscoring limitations in signal-dependent spatial reasoning [23].

Importantly, most of these studies were conducted in hospital or emergency populations with higher disease prevalence and more conspicuous ECG abnormalities. In contrast, our study was performed in a low-prevalence disease screening population characterized by high physiological variability and frequent borderline patterns. In this setting, discrimination depends on subtle deviations from exercise-induced remodeling rather than overt pathological features. The observed AUC of 0.52 in our cohort is therefore consistent with, and in some cases lower than, previously reported LLM performance in acute-care settings, suggesting that probabilistic image-level reasoning without task-specific training is insufficient for reliably discriminating cardiovascular disease in competitive athletes.

### 4.3. Borderline ECGs: The Critical Failure Zone in PPS

The misclassification analysis provides clinically relevant insight.

False-negative cases were overwhelmingly characterized by borderline ECG patterns (82%) according to International Criteria [3]. Borderline findings—such as isolated axis deviations or atrial enlargement—often lie at the interface between physiological adaptation and early pathological remodeling in athletes [2]. These patterns require contextual interpretation and, in selected cases, targeted imaging [5].

Notably, the model also failed to identify a substantial number of athletes with overt red-flag ECG abnormalities. This indicates that the observed performance limitations were not confined to subtle presentations but extended to clearly pathological patterns.

In PPS, the primary objective is risk mitigation in a low-prevalence population [24]. Failure to detect borderline or overt abnormalities may generate false reassurance in precisely those cases where careful clinical evaluation is warranted.

### 4.4. Clinical Relevance in a Low-Prevalence Screening Setting

Predictive values must be interpreted in light of disease prevalence. In PPS populations, where the prevalence of clinically relevant cardiovascular disease is relatively low, even poorly discriminative tools may yield apparently acceptable negative predictive values due to class imbalance [24].

In our study, the NPV largely reflected the model’s systematic tendency to assign near-zero scores combined with the high proportion of non-diseased athletes, rather than robust discriminatory capacity. This distinction is critical in screening settings, where the goal is not probabilistic stratification but reliable identification of individuals requiring further evaluation.

Given that established athlete-specific ECG interpretation criteria already aim to maximize sensitivity while reducing false positives, the addition of a poorly discriminative probabilistic score does not currently provide incremental clinical value.

### 4.5. Implications for AI Use in Sports Cardiology

Our findings should not be interpreted as evidence against AI in ECG interpretation. On the contrary, signal-based deep learning approaches have demonstrated clinically meaningful performance in detecting structural heart disease and ventricular dysfunction, and growing evidence supports the potential role of AI in sports cardiology [8].

However, our results indicate that general-purpose LLMs, when used without task-specific training or calibration, should not be relied upon for diagnostic decision-making or triage in ECG-based PPS. The increasing accessibility of LLMs may encourage informal exploratory use in clinical practice. In the absence of validation, such use could inadvertently influence interpretation or downstream testing decisions. At present, the appropriate role of LLMs in sports cardiology is likely supportive rather than diagnostic—for example, assisting with documentation, structured reporting, or educational tasks—while disease detection should remain grounded in expert interpretation and validated signal-based AI tools [8].

### 4.6. Limitations and Future Directions

First, we intentionally evaluated a general-purpose LLM in a naïve, non-task-trained configuration to reflect real-world accessibility rather than optimized performance [13,14,15]. Although prompt engineering, domain-specific fine-tuning, or integration of structured clinical data could potentially improve performance, such approaches would effectively transform the system into a task-specific AI model rather than a general-purpose LLM. Therefore, our findings should be interpreted within the context of unsupervised, real-world use.

Second, we did not include a direct comparison with expert ECG interpretation or dedicated signal-based AI models [7,8]. As a result, our study does not provide a relative performance benchmark but rather an absolute evaluation of LLM performance in this setting.

Third, not all cardiovascular conditions included in PPS are expected to be detectable from ECG alone [2]. However, this limitation was addressed through a predefined sensitivity analysis restricted to ECG-detectable conditions. The persistence of poor discrimination in this subset suggests that the observed performance limitations cannot be explained solely by the inclusion of non-ECG-detectable diseases.

Fourth, the prevalence of confirmed cardiovascular disease in our cohort was higher than typically observed in unselected PPS populations [24]. This likely reflects the multicentre and clinically enriched nature of the dataset, including athletes undergoing second- and third-line investigations. While this may limit generalizability, it also reflects real-world screening pathways.

Fifth, ECG image quality and acquisition variability across centers may have introduced noise. However, this variability mirrors real-world PPS conditions and may enhance external validity.

Finally, the analysis was conducted using a specific commercially available version of an LLM. Given the rapid evolution of such models, performance may vary across versions and over time [13,14,15].

Future research should explore domain-adapted multimodal architectures trained directly on athlete-specific ECG datasets, integrating signal-level learning with contextual clinical features [8]. Until such models are prospectively validated, screening decisions in competitive athletes should continue to rely on established interpretation criteria and specialist evaluation [1,3].

## 5. Conclusions

In this multicentre PPS cohort, a general-purpose LLM used in a naïve configuration demonstrated no meaningful discriminatory ability for cardiovascular disease from athlete ECGs. Performance was close to random classification and failures predominantly involved borderline ECG patterns.

These findings suggest that, in its current form, a commercially available LLM should not be used for diagnostic triage in athlete screening. ECG-based PPS should continue to rely on established interpretation criteria and validated signal-based AI tools.

## Figures and Tables

**Table 1:** Baseline Characteristics of the Study Population (N = 2950).

Variable	Value
Age (years)	23 ± 9
Male sex	72%
Confirmed cardiovascular disease	450 (15.3%)
No disease	2500 (84.7%)

**Table 2:** Distribution of Confirmed Cardiovascular Diagnoses (n = 450).

Diagnosis	n	%
Mitral valve prolapse	80	17.8%
Hypertension	52	11.6%
Bicuspid aortic valve	47	10.4%
Atrial septal defect	35	7.8%
Non-ischemic left ventricular scar	33	7.3%
Coronary artery anomalies	32	7.1%
Wolff–Parkinson–White	25	5.6%
Patent foramen ovale	25	5.6%
Hypertrophic cardiomyopathy	21	4.7%
Left ventricular non-compaction	14	3.1%
Long QT syndrome	12	2.7%
Ischemic heart disease	10	2.2%
Dilated cardiomyopathy	8	1.8%
Ventricular septal defect	7	1.6%
Arrhythmogenic cardiomyopathy	7	1.6%
Moderate aortic regurgitation	7	1.6%
Pericarditis	6	1.3%
Catecholaminergic polymorphic ventricular tachycardia	5	1.1%
Aortic stenosis	5	1.1%
Myocarditis	5	1.1%
Moderate mitral regurgitation	5	1.1%
Pulmonary valve stenosis	4	0.9%
Brugada syndrome	2	0.4%
Anomalous pulmonary venous return	2	0.4%
Transposition of great vessels	1	0.2%

**Table 3:** Diagnostic Performance at Youden-Derived Threshold (GPT ≥ 0.05).

Metric	Value
True positives	93
False negatives	357
True negatives	1675
False positives	825
Sensitivity	20.7%
Specificity	67.0%
PPV	10.1%
NPV	82.4%
AUC	0.52 (95% CI 0.49–0.55)

**Table 4:** International ECG Criteria Distribution in Diseased Athlete.

ECG Category	True Positives (n = 93)	False Negatives (n = 357)
Red	42 (45.2%)	56 (15.7%)
Yellow	26 (28.0%)	294 (82.4%)
Green	25 (26.9%)	7 (2.0%)

## Data Availability

Data are available upon reasonable request on corresponding author.
